# Mumps Cases Disproportionately Affecting Persons Living with HIV Infection and Men Who Have Sex with Men — Chicago, Illinois, 2018

**DOI:** 10.15585/mmwr.mm6928a3

**Published:** 2020-07-17

**Authors:** Tristan D. McPherson, Enrique Ramirez, Madeline Ringness, Peter Ruestow, Mariel Marlow, Marielle J. Fricchione

**Affiliations:** ^1^Epidemic Intelligence Service, CDC; ^2^Communicable Disease Program, Chicago Department of Public Health; ^3^Immunization Program, Chicago Department of Public Health; ^4^School of Health Professions, University of Missouri-Columbia; ^5^Division of Viral Diseases, National Center for Immunization and Respiratory Diseases, CDC.

During January 1–March 2, 2018, the number of mumps cases among adults reported to the Chicago Department of Public Health (CDPH) doubled compared with the same period in 2017. In response, CDPH created a supplementary questionnaire to collect additional information on populations affected and potential transmission routes. An epidemiologic analysis of routine and supplementary data, including spatiotemporal analysis, was performed to describe mumps cases reported to CDPH during 2018. A fourfold increase in mumps cases was reported during 2018 compared with 2017, with men who have sex with men (MSM) and persons living with human immunodeficiency virus (HIV) infection disproportionately represented among cases. A spatiotemporal, residential cluster was identified in a 9–square-mile area within six adjacent communities. The majority of persons affected were MSM, and this area was visited by many other persons with mumps diagnoses. Spatiotemporal analyses could be used in real time to identify case clusters to target public health response efforts, including to guide recommendations for additional measles, mumps, and rubella (MMR) vaccine and to identify specific transmission venues.

## Investigation and Findings

During January–March 2018, 14 confirmed or probable mumps cases (*1*) were reported to CDPH. Four cases were among children (aged <18 years), consistent with case counts among children from previous years, and 10 were among adults aged ≥18 years. Although most mumps outbreaks among adults occur in university settings (*2*), none of the 14 patients reported university exposure. Providers had voluntarily reported data on MSM and HIV status on many early patients, although this information was not systematically requested. By April 2018, a total of 23 mumps cases among adults had been reported, including 11 (48%) among MSM, and of these, five were persons living with HIV infection. CDPH undertook an epidemiologic investigation of adults with mumps to identify specific populations affected and transmission settings to more effectively target public health response efforts.

Standard case investigation questionnaires focusing on demographic characteristics, signs and symptoms, and school or university exposures were used; however, because of case predominance among adults and MSM, in April 2018, CDPH developed a supplementary questionnaire with input from the CDPH sexually transmitted infection (STI) division (*3*). That questionnaire focused on adult-specific settings, including gyms, bars, clubs, bathhouses, sex parties, or concerts; and activities such as sharing smoking products or drinks, online dating application meetings, and sexual contact. Both questionnaires were administered to adults who received mumps diagnoses in 2018. The supplementary questionnaires were administered retrospectively to persons with mumps reported to CDPH before April 2018. HIV infection status and most recent available CD4+ count were ascertained through direct provider report or the Enhanced HIV/AIDS Reporting System,* a CDC application that assists health departments with reporting, data management, and analysis. Data from adults with mumps reported during 2018 were analyzed, excluding adults for whom the standard questionnaire was not completed.

SaTScan (version 9.6; https://www.satscan.org), a free software program for analysis of spatial, temporal, and space-time data, was used in January 2019 for retrospective spatiotemporal cluster detection by patient residence, using a discrete space-time Poisson model. Fisher’s exact test was used to identify differences in prevalence estimates using SAS (version 9.4; SAS Institute). Mumps virus genotyping was performed by CDC on mumps patients’ buccal swabs or urine samples, and these sequences were compared to identify any differences that might indicate that cases were not related and that more than one outbreak was occurring.

**FIGURE 1:**
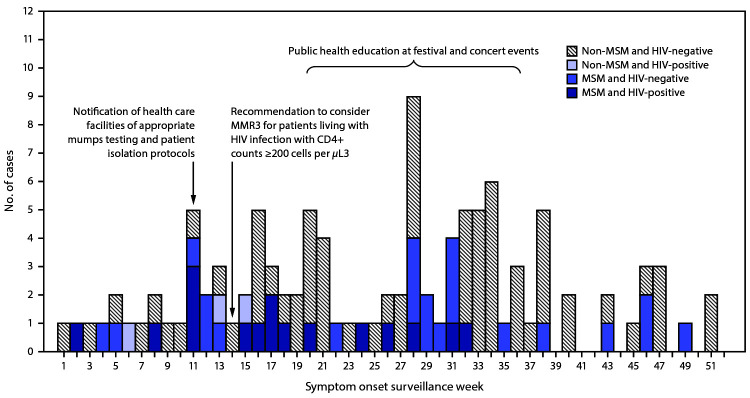
Number of mumps cases among adults aged ≥18 years (N = 110),* by sexual practice, human immunodeficiency virus (HIV) status, and week of symptom onset — Chicago, January–December 2018 **Abbreviations:** MMR3 = 3rd dose of measles, mumps, and rubella vaccine; MSM = men who have sex with men. * Excludes six patients who could not be contacted.

After April 2018, an additional 93 confirmed and probable mumps cases among adults were reported to CDPH, for a total of 116 during January–December 2018. Median patient age was 29 years (interquartile range = 26–38 years). Standard questionnaire data were available from 110 (95%) persons (Figure 1). Among these 110 patients, 76 (69%) were male (Table). Overall, 101 (92%) persons reported having received at least 1 dose of MMR vaccine, although only nine reports could be confirmed through vaccination records. Five patients reported a university exposure; two of these cases were associated with a known university outbreak that occurred outside of Chicago.

**TABLE:** Characteristics of mumps cases among adults aged ≥18 years* — Chicago, Illinois, January–December 2018

Characteristic	No. (%)
**Age, median, years (IQR)**	29 (26–38)
**Gender**	
Male	76 (69)
Female	33 (30)
Transfeminine^†^	1 (1)
**Race/Ethnicity**	
White, non-Hispanic	53 (48)
Black, non-Hispanic	32 (29)
Hispanic	21 (19)
Asian, non-Hispanic	3 (3)
Unknown	1 (1)
**Complications**	
Trouble hearing	71 (65)
Oophoritis^§^	4 (12)
Mastitis^§^	4 (12)
Orchitis^¶^	22 (29)
Hospitalization**	13 (37)
**Sexual partner history^††^**	
Men who have sex with men	37 (34)
Women who have sex with men	29 (31)
Men who have sex with only women	27 (29)
**Potential exposure locations^††^**	
Bars	57 (84)
Gyms	38 (56)
Concerts	29 (28)
Online dating application meetings	15 (22)
Bathhouses	4 (6)

**Abbreviation:** IQR = interquartile range.

* N = 110. Excludes six patients who could not be contacted.

^†^ A person assigned male sex at birth, but who identifies with femininity to a greater extent than with masculinity.

^§^ Among 33 women.

^¶^ Among 76 males and one transfeminine person.

** The most common reasons for hospital admission included empiric antibiotic treatment because of concern for bacterial infection (three), intravenous hydration and pain control (two), and request for evaluation by otolaryngologists or infectious disease physicians (two).

^††^ Among those with supplementary data available (N = 93).

Complications reported included trouble hearing (71; 65% patients); lower abdominal pain, a symptom consistent with oophoritis (four; 12% of 33 female patients), and symptoms consistent with mastitis (four; 12% of 33 female patients). Among 76 males and one transfeminine^†^ person, 22 (29%) reported orchitis; 13 (59%) orchitis cases were provider-confirmed.

Nineteen (17%) mumps patients were persons living with HIV infection, including 13 (68%) with CD4+ counts available within 18 months before onset of mumps symptoms; 12 (63%) persons had values ≥200 cells *μ*L^3^. There were no differences in complications among persons living and not living with HIV infection. No person with a reported mumps case named others with a reported mumps case as a contact.

Overall, 35 (32%) mumps diagnoses occurred in emergency departments; 13 (37%) patients required hospitalization. There was a significant difference in the prevalence of hospitalization of mumps patients living with HIV infection (six of 19; 32%) and those who were not (seven of 91; 8%) (p = 0.01). Reasons for hospitalization varied (Table).

Among 110 patients with standard questionnaire data available, supplementary questionnaire data were available for 93 (85%); 29 (31%) reported being women who have sex with men, 27 (29%) as men who have sex with only women, and 37 (34%) as MSM. Among 19 mumps patients living with HIV infection, 16 (84%) were MSM. Sixty-eight (73%) patients reported recent potential exposure to one or more specific close-contact environments (Table).

**FIGURE 2:**
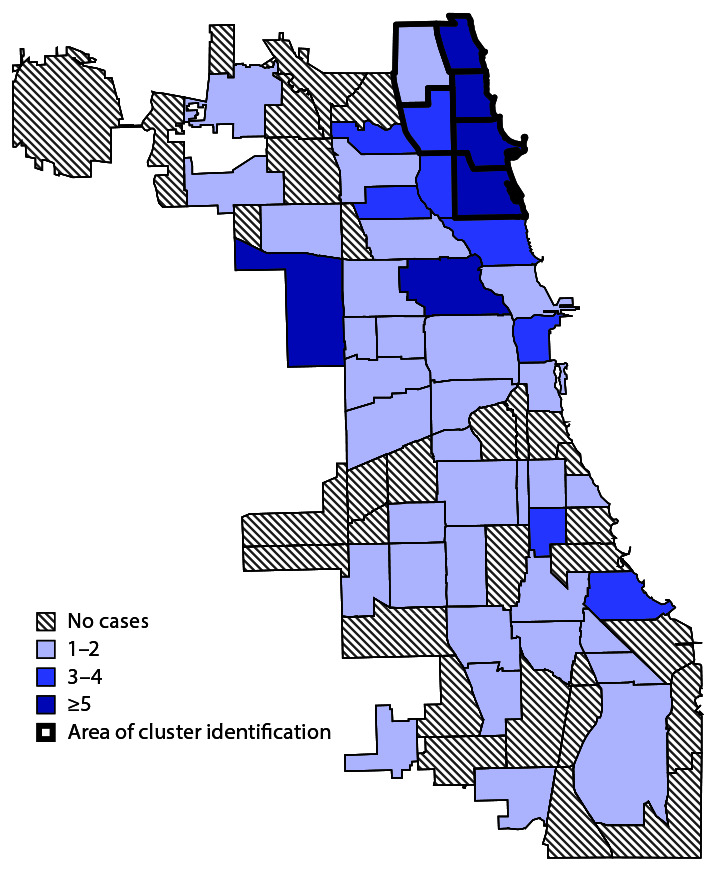
Number of mumps cases among adults aged ≥18 years (N = 110),* by community area^†^ — Chicago, Illinois, January–December 2018 * Excludes six patients who could not be contacted. ^†^ Chicago is divided into 77 community areas whose boundaries do not change over time (https://www.chicago.gov/city/en/depts/dgs/supp_info/citywide_maps.html).

Among patients with supplementary questionnaire data, a spatiotemporal cluster was identified that included 10 patients with home addresses in a 9–square-mile area within six adjacent communities (Figure 2). Among those 10 persons, eight were MSM, seven visited multiple bars near their homes, and two were bartenders or servers at these bars during their infectious period (from 2 days before until 5 days after parotitis onset). Eighteen other persons with residences geographically dispersed throughout the city developed mumps after visiting bars in the cluster area, and one additional bartender was identified who worked while infectious but did not provide names or locations of workplaces. Overall, 65 mumps patients were either not temporally related to these 10 cases or were geographically dispersed and did not report visiting close-contact environments in the cluster area. Genotype results were available for six patients; all demonstrated the most common nationally circulating sequence [MuVi/Sheffield.GBR/1.05 (G)], which provided no additional information on epidemiologic links.

## Public Health Response

On March 16, 2018, CDPH released a citywide health alert notifying health care providers of increased mumps reports among adults and provided instructions to confirm mumps using polymerase chain reaction testing. On April 6, CDPH released recommendations and provided education to clinics and health care facilities to administer MMR vaccine to any patients or health care workers without evidence of immunity,^§^ reinforce infection control measures, and offer a third MMR dose to persons living with HIV infection whose CD4+ counts were ≥200 cells *μ*L^3^ because of initial concern for increased risk of complications (*4*–*6*).

Throughout 2018, CDPH worked with city partners to promote education and awareness of mumps during citywide Pride events. CDPH considered recommending targeted vaccination campaigns among specific populations most affected by illness; however, in the absence of real-time cluster detection analysis and supplementary questionnaire data, CDPH was not readily able to identify additional groups at increased risk who should receive additional MMR doses (*4*).

## Discussion

In 2018 in Chicago, the 116 mumps cases among adults exceeded the number observed during the previous 5 years combined (80); transmission continued throughout 2018, despite infection control activities. MSM and persons living with HIV infection were disproportionately affected, accounting for 34% and 17% of cases, respectively, despite these groups representing 5% and <1% of Chicago’s population, respectively (*7*,*8*). Cases were widely dispersed across the city. In addition, the proportion of persons reporting complications was higher than that in previous studies (*2*), although typically, these data are limited by their self-reported nature, except for orchitis. As of August 30, 2019, only 11 mumps cases had been reported to CDPH during 2019, indicating a return to baseline activity.

Most recent mumps outbreaks in the United States have occurred at university campuses and other settings where targeted vaccination campaigns can be conducted (*2*). Mumps cases in Chicago during 2018 demonstrate challenges in identifying and containing clusters when persons are geographically dispersed and report multiple close-contact behaviors. Using spatiotemporal analyses, a residential cluster of patients who shared a common exposure (bars in a 9–square-mile area) was retrospectively identified, indicating this approach could be used for mumps investigations. Had spatiotemporal analyses been available in real time, the identified cluster could have been recognized earlier, providing an opportunity to target control measures within a defined location or social network including possible recommendations for a third MMR dose for persons frequenting or working at specific venues. This investigation successfully used a supplementary questionnaire, demonstrating that persons might be willing to provide sensitive information to facilitate public health interventions related to mumps.

As a result of this investigation, CDPH is developing protocols to use spatiotemporal analysis in real time to more rapidly identify clusters of vaccine-preventable diseases, including mumps. CDPH continues to investigate adults with mumps cases using the supplementary questionnaire to determine epidemiologic links and guide recommendations for additional MMR doses.

**Summary**

**What is already known about this topic?**

The majority of mumps cases among U.S. adults has been reported in university settings with a readily identified target population for outbreak response.

**What is added by this report?**

This report describes increased mumps cases in a nonuniversity setting with geographical distribution throughout a large urban center, disproportionately affecting men who have sex with men and persons living with human immunodeficiency virus infection. Challenges in determining transmission settings and effective response plans were identified.

**What are the implications for public health practice?**

This investigation highlights the use of spatiotemporal analysis to identify mumps clusters in real time as a tool for targeted outbreak interventions and ability to collect potentially sensitive data in the context of adult-specific exposure locations outside of university settings.

## Footnotes

* https://www.cdc.gov/hiv/library/reports/hiv-surveillance.html.

† A person assigned male sex at birth, but who identifies with femininity to a greater extent than masculinity.

§ Evidence of immunity includes 1) written documentation of receipt of 1 dose of a mumps-containing vaccine administered on or after the first birthday for preschool-aged children and adults not at high risk, and 2 doses of mumps-containing vaccine for school-aged children and adults at high risk (i.e., health care personnel, international travelers, and students at post high school educational institutions); 2) laboratory evidence of immunity; 3) laboratory confirmation of disease; or 4) birth before 1957.
